# Effects of iron supplementation on growth, gut microbiota, metabolomics and cognitive development of rat pups

**DOI:** 10.1371/journal.pone.0179713

**Published:** 2017-06-29

**Authors:** Erica E. Alexeev, Xuan He, Carolyn M. Slupsky, Bo Lönnerdal

**Affiliations:** 1 Department of Nutrition, University of California, Davis, CA, United States America; 2 Department of Food Science and Technology, University of California, Davis, CA, United States America; University of Illinois at Urbana-Champaign, UNITED STATES

## Abstract

**Background:**

Iron deficiency is common during infancy and therefore iron supplementation is recommended. Recent reports suggest that iron supplementation in already iron replete infants may adversely affect growth, cognitive development, and morbidity.

**Methods:**

Normal and growth restricted rat pups were given iron daily (30 or 150 μg/d) from birth to postnatal day (PD) 20, and followed to PD56. At PD20, hematology, tissue iron, and the hepatic metabolome were measured. The plasma metabolome and colonic microbial ecology were assessed at PD20 and PD56. T-maze (PD35) and passive avoidance (PD40) tests were used to evaluate cognitive development.

**Results:**

Iron supplementation increased iron status in a dose-dependent manner in both groups, but no significant effect of iron on growth was observed. Passive avoidance was significantly lower only in normal rats given high iron compared with controls. In plasma and liver of normal and growth-restricted rats, excess iron increased 3-hydroxybutyrate and decreased several amino acids, urea and *myo*-inositol. While a profound difference in gut microbiota of normal and growth-restricted rats was observed, with iron supplementation differences in the abundance of strict anaerobes were observed.

**Conclusion:**

Excess iron adversely affects cognitive development, which may be a consequence of altered metabolism and/or shifts in gut microbiota.

## Introduction

Iron deficiency is the most prevalent nutrient deficiency worldwide [1]. Infants are the most vulnerable population and exhibit the highest prevalence of iron deficiency anemia (IDA), which is associated with long lasting neurocognitive impairments [2, 3]. While iron supplementation or fortification is generally effective for preventing IDA, several studies suggest that providing iron to infants who are iron replete may have adverse consequences [4–8]. Daily ferrous sulfate supplementation (1 mg/kg) to iron-sufficient breast-fed infants in Sweden and Honduras resulted in significantly lower length gain and head circumference compared to placebo [5]. Similarly, Ziegler et al. [9] showed that breast-fed infants in the U.S. given iron drops from 4 to 9 months of age had significantly lower length gain and a trend towards lower weight gain than control groups. Further, a group of Italian researchers showed that breast-fed infants given oral iron supplements from 4 to 9 months of age had significantly shorter length at 12 and 18 months of age than unsupplemented breast-fed infants or infants fed iron-fortified formula [10]. In contrast, studies on iron supplementation to iron-replete infants from less developed countries have only shown an adverse effect on weight gain, but not length [5, 7, 8]. Since the nutritional status of these infants was sub-optimal, it is speculated that the iron-induced growth effect was manifested by a decrease in weight gain.

Excess iron during infancy has also been shown to have a negative long-term impact on neurodevelopmental outcomes. A 10-year-long follow up study by Lozoff et al. showed that consumption of iron-fortified infant formula (with 10.6 mg/L of iron as ferrous sulfate) by 6 months old infants with initially high hemoglobin levels resulted in lower scores on IQ, spatial memory, and visual-motor integration at 10 years of age compared with those who were fed low (2.8 mg/L) iron formula [6]. In our study on Swedish and Honduran infants, a beneficial effect of iron on diarrhea in infants with low hemoglobin was observed, whereas iron-replete infants given iron had more diarrhea than infants given placebo [5]. A large intervention study by Soofi et al. [11] showed that iron supplied in a micronutrient powder given alone or with zinc daily to 6 to 18 months old Pakistani children reduced IDA in both groups, but resulted in a significantly higher incidence and proportion of days with diarrhea than in controls. Moreover, a systematic review of iron supplementation trials on children showed an increased risk of developing diarrhea [12]. Unfortunately, our knowledge of the mechanisms underlying the adverse effects of iron on infant growth and development is very limited.

With respect to the microbiome, it has recently been shown that an iron-fortified micronutrient powder given to Kenyan infants from 6 to 10 months of age resulted in increased enterobacteria, especially pathogenic *E*. *coli*, and decreased bifidobacteria, which was accompanied by a significantly larger number of infants requiring treatment for diarrhea than controls [13]. The authors also noted significantly higher levels of calprotectin in infants given iron, which is indicative of intestinal inflammation. These observations suggest that iron-induced changes in the gut microbiota may be a factor in infant morbidity. Whether alterations of the gut microbiota can affect physical growth and/or cognitive development is not yet known.

Given the clinical evidence of adverse effects of excess iron given to infants, we investigated the interaction between iron supplementation in the context of a nutritionally adequate and inadequate diet. Using a previously established rat pup model of caloric restriction [14], effects of two levels of iron supplementation on growth, cognitive development, plasma and liver metabolic profiles, and gut microbiota were measured in normal and growth-restricted rat pups. We hypothesized that energy and macronutrient intake may affect the outcome of iron supplementation.

## Materials and methods

### Experimental design

Fifteen pregnant Sprague Dawley rats were obtained from Charles River (Wilmington, MA) at 14 days of gestation and maintained on a standard, non-purified rodent diet (LabDiet 5001, Purina, Hayward, CA) in solid plastic hanging cages under constant conditions (temperature, 22°C; humidity, 62%) with a 12-h light-dark cycle. All offspring were randomized at birth and assigned into sex-matched litters of either 10 pups (Normal, n = 60) or litters of 18 pups (Growth restricted, n = 72) and nursed by their respective dams.

On postnatal day (PD) 2, pups from the normal or growth restricted litters were randomly assigned to three groups followed by daily gavage until PD20 of: (1) 25 μL of a 10% sucrose solution (Control, n = 44); (2) 30 μg Fe as ferrous sulfate (Sigma, St. Louis, MO) in 25 μL 10% sucrose solution (medium-Fe group, n = 44); or (3) 150 μg Fe as ferrous sulfate in 25 μL 10% sucrose solution (high-Fe group, n = 44) as previously described [15]. These iron levels were chosen to be similar on a body weight basis to human infants receiving iron drops or iron-fortified formula (16). Ferrous sulfate solutions were prepared fresh daily prior to supplementation. Pups were identified using a micro-tattooing system (Fine Science Tools Inc., Foster City, CA) to allow identification of pups while minimizing stress to the animals. In the normal group, 3–4 animals were assigned to each Fe or control treatment group, while in growth-restricted litters, 6 animals were assigned to each Fe or control treatment group. Weights were monitored every other day from PD2 to PD56. All pups were weaned at PD21 to a standard, non-purified rodent chow (LabDiet 5001, Purina, Hayward, CA), fed *ad libitum*, and housed with 3–5 animals per cage. Rats were euthanized on PD20 and PD56, and blood, colon, liver and spleen were collected. Liver and spleen tissue were flash-frozen in liquid nitrogen and stored at -80°C. Blood samples were drawn into EDTA tubes and plasma was collected after centrifugation. Samples were frozen and stored at -20°C until analysis. All animal procedures were approved by the University of California Institutional Animal Care and Use Committee.

### Blood measurements

Whole blood hemoglobin was measured by the cyanmethemoglobin method using a commercially available kit (Sigma, St. Louis, MO). For hematocrit measurement, whole blood was collected in heparinized capillary tubes (Fisher Scientific, Pittsburgh, PA), centrifuged and placed in a hematocrit reader.

### Tissue Fe measurement

Liver and spleen samples were dissected and digested in HNO_3_ (16 mol/L) for 7 d and then at sub-boiling temperatures for 6–8 h [16]. Samples were diluted with deionized water and analyzed for iron by atomic absorption spectrometry (model Smith-Heifjie 4000, Thermo Jarrell Ash Corporation, Franklin, MA).

### T-maze

Spatial memory and learning were measured by spontaneous alternation in a T-maze at PD35. In the T-maze test, rats were tested on their capability to alternate between two directions of an enclosed apparatus in the form of a T placed horizontally, as previously described [17]. Upon successful alternation of direction, animals were given a score of 1. This was repeated ten times, with the maximum score being 9.

### Passive avoidance

Memory and learning were examined by the passive avoidance test at PD40. Passive avoidance testing was carried out in animals using an apparatus consisting of two Perspex boxes (30 cm × 20 cm × 20 cm) separated by a removable door. One of the boxes was constructed of black Perspex (dark chamber), and the other white, opaque Perspex and open to the light (light chamber). An opening with a removable door separated the chambers. The floor of the dark chamber was composed of a metal grid through which a small electrical charge (2.0 mA) could be passed. Testing was carried out over 2d: a training (habituation) day and a testing (learning) day as previously described [18, 19]. Data are expressed as time (s) taken to enter the dark chamber on d2 of the test, designated as retention latency. For the purpose of this test, longer retention latency indicates the animal remembers the shock administered the previous day.

### Hepatic metabolite extraction

Liver tissue from PD20 (weight: 195.6 ± 3.1 mg) was homogenized by bead beating with 1.4 mm ceramic spheres beads (Lysing Matrix D, MP Biomedicals, Solon, OH) using an MP FastPrep bead beater (FastPrep-24 homogenizer, MP Biomedicals, Solon, OH) at a speed of 6.0 m/s for 60 s. The liver tissue homogenates were then extracted using the Bligh and Dyer method [20] with the following modifications. After adding 100 μL of ice cold Milli-Q water, homogenized samples were thoroughly mixed with 4 volumes of methanol-chloroform (2:1 v/v), followed by addition of one part of chloroform and one part of Milli-Q water. After 10 min at room temperature, samples were centrifuged at 100 *x g* for 15 min. 450 μL of the top methanol/water layer of each sample was carefully collected and lyophilized using a Labconco FreeZone 4.5 L Freeze Dry System (Labconco; Kansas City, USA). Lyophilized samples were diluted in 300 μL of phosphate buffer (4.7 mM K_2_HPO_4_, 5.3 mM KH_2_PO_4_, pH 6.85) and centrifuged at 14k *x g* at 4°C for 10 min. Supernatant (207 μL) was mixed with 23 μL of internal standard containing 5 mmol/L 3-(trimethylsilyl)-1-propanesulfonic acid-d_6_ (DSS-d_6_), and samples were prepared for analysis as described below.

### NMR-based metabolome profiling

EDTA plasma samples were removed from -20°C storage and allowed to thaw, after which they were filtered through a 3,000 MW cutoff filter (Amicon, 0.5 mL capacity, Milipore) to remove insoluble lipid particles and proteins. If necessary, an appropriate amount of Milli-Q water was added to the filtrate to achieve a volume of 199 μL, followed by 8 μL of potassium phosphate buffer (1 M, pH 6.1), and 23 μL of internal standard containing 5 mM DSS-d6, 0.2% NaN_3_ in 99.8% D_2_O. A final volume of 180 μL was transferred to a 3 mm NMR tube. Samples were stored at 4°C until NMR data acquisition. NMR spectra were acquired at 25°C using the Bruker noesypr1d experiment on a Bruker Avance 600 MHz NMR spectrometer. ^1^H NMR spectra were acquired with a spectral width of 12 ppm, a total acquisition time of 2.5 s, 8 dummy scans, 32 transients and water saturation during the prescan delay (2.5 s) and mixing time (100 ms) to minimize impact of water on the spectrum as previously described [21]. NMR spectra were zero-filled to 128k data points, Fourier Transformed with a 0.5-Hz line broadening, and manually phased and baseline corrected using Chenomx NMR Suite v8.0 Processor (Chenomx Inc., Edmonton, Canada). Profiler was used to quantify metabolites using the concentration of the reference signal (DSS-d6) as previously described [21]. Concentrations are reported in micromol/L (μM).

### 16s rRNA microbial profiling

Contents from the distal colon obtained at the time of sacrifice on PD20 (n = 36) and PD56 (n = 24) were subjected to 16s rRNA microbial profiling. Prior to DNA extraction, samples were randomized to ensure no bias in sample preparation. Qiagen QIAamp DNA Stool Mini Kit (QIAGEN, Hilden, Germany) was used for DNA extraction according to the manufacturer’s standard protocol with the following modifications: after addition of ASL buffer, samples were subjected to bead beating for 2 min followed by heating for 5 min at 95°C. PCR was used to amplify the V4 region of the 16S rRNA gene from each DNA sample using the F515/R806 primer. An 8 bp Hamming error-correcting barcode was used to enable sample multiplexing. The PCR amplicons were purified using the QIAquick PCR purification kit (QIAGEN, Hilden, Germany) according to manufacturer’s instructions. Purified DNA isolates were submitted to the UC Davis Genome center DNA technologies core for 250 bp paired-end sequencing on the Illumina Miseq platform.

The pair-ended sequences were analyzed using Quantitative Insights Into Microbial Ecology (QIIME) pipeline (version 18.0) [22] based on the close reference OTU selection method. Briefly, OTU selection was performed using UCLUST [23] against the most recent version of the Greengenes core database (“gg_13_8_otus”) and clustered with a threshold of 97% identity excluding reads that did not match the database. These data were refracted by the minimum observation count. Principal coordinates analysis (PCoA) of unweighted UniFrac distance [24] was computed to illustrate the relationship between the bacterial β-diversity from different diets (normal vs. restricted diet) and iron dosage (control, medium, and high) and visualized using EMPeror [25]. α-diversity (observed species) was computed within QIIME.

For analysis of facultative to strict anaerobes ratio, a method developed by Arboleya et al. [26] was modified as follows. Briefly, the proportions of facultative and strict anaerobes were estimated by the sum of the corresponding microbial group and normalized by the total percentage of microbes so that % of all strict and facultative anaerobes was 100%. The facultative anaerobes included the family of Enterobacteriaceae and Enterococcaceae, as well as the genera of *Lactobacillus*, *Streptococcus*, *Staphylococcus*, *Jeotgalicoccus*, *Aerococcus* and *Facklamia*. The strict anaerobes included the genera of *Bifidobacterium*, *Bacteroides*, *Clostridium* (from both family of Clostridiaceae, Erysipelotrichaceae and Lachnospiraceae), *Prevotella*, *Turicibacter*, *Sarcina*, *Blautia*, *Coprococcus*, *Eubacterium*, *Ruminococcus* and *Akkermansia*.

### Statistical analysis

For analysis of cognitive score, as well as anthropometric and biochemical measurements: Values are presented as means ± SEM, unless otherwise noted. The effect of litter size and iron supplementation on pup weight was assessed by repeated measures ANOVA. When ANOVA showed a significant effect, differences between groups were assessed using post-hoc Tukey’s test. Subgroup analysis was done using Student’s *t-*test where noted. All statistical analyses were performed using GraphPad Prism (Version 5.0, GraphPad Software, San Diego, CA) or R. Statistical significance was accepted at *p* < 0.05.

Metabolomics and microbiome data: Metabolomics data were log transformed and microbial relative abundance data were arcsine transformed to approximate normal distribution. Principal components analysis (PCA) was performed using R and visualized using ggplot2. Individual metabolite variables and bacterial relative abundance were analyzed using two-way ANOVA (iron treatment X diet for PD20 and PD56) and one-way ANOVA (for each diet group) followed by post-hoc Tukey’s test in R. The differences between PD20 and PD56 in each Fe treatment and diet group were assessed using Student’s t-test.

## Results

### Growth and iron status assessment

**Fig 1:**
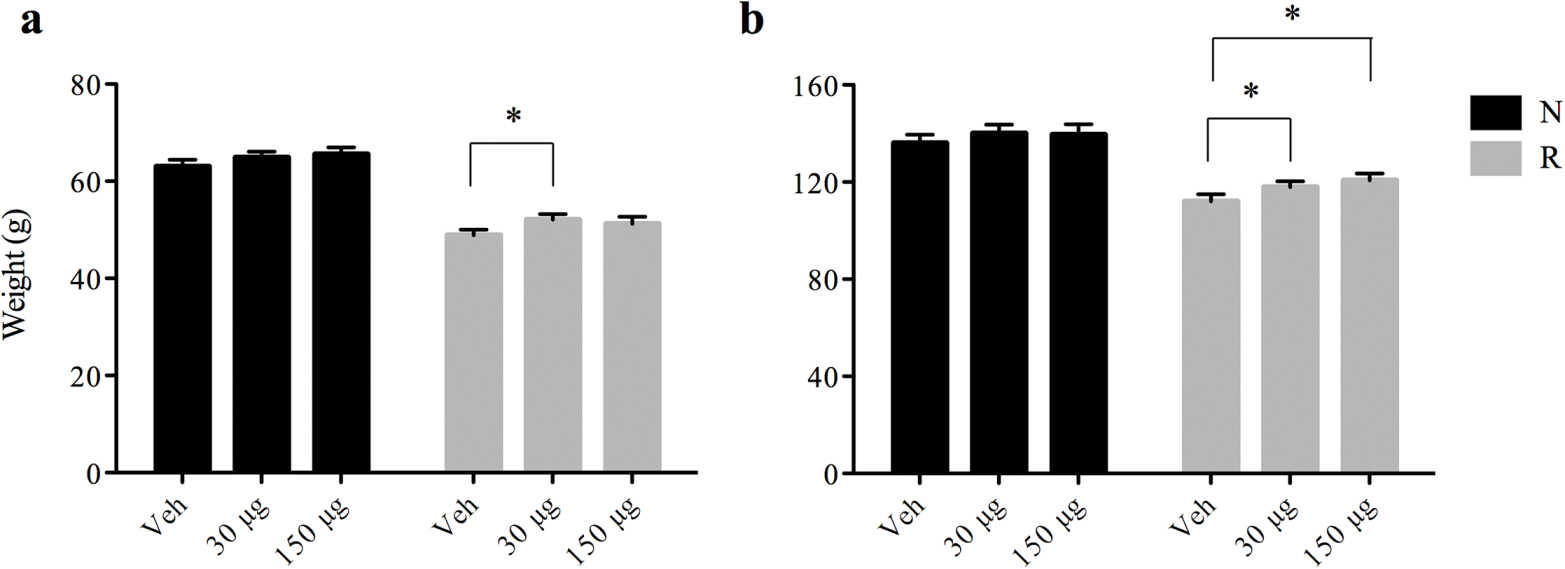
Effect of iron supplementation on weight at PD22 and PD32. (**a**) At PD22 the weight of pups receiving 30 μg of Fe was significantly higher (52.09 ± 0.19 g vs. 48.87 ± 1.1 g, *p* < 0.05) than controls (Veh) in growth-restricted rats. (**b**) At PD32, the weights of the pups receiving 30 μg and 150 μg of Fe/day were significantly larger than those receiving vehicle (Veh) (*p* < 0.05) using one-way ANOVA. Data are presented as means ± SEM. In black are normal rats, and in grey are growth-restricted rats.

At birth, pups were randomized into normal (N) or restricted (R) growth litters and administered vehicle, 30 μg Fe, or 150 μg Fe daily from PD2-20. After randomization, there was no significant difference in weight at PD2 (t-test). However, a significant reduction in body weight in the growth restricted rats as early as PD4 (11.22 ± 0.16 g vs. 9.66 ± 0.11 g, (t-test, *p* < 0.001) and in the overall trajectory before weaning (PD2 –PD20, repeated measures ANOVA, *p* < 0.05) compared to normal growth rats was observed. The delayed growth due to early growth restriction was recovered after weaning with an energy adequate diet. There was no significant effect of iron supplementation on growth during the supplementation period (PD2 to PD20) regardless of dosage or energy intake (repeated measures ANOVA). Interestingly, during the post-iron supplementation period with normal rat chow, there was a slight improvement in the weight of rats in the growth-restricted medium iron treatment group at PD22 compared to the corresponding control group (52.09 ± 0.19 g vs. 48.87 ± 1.1 g, Fig 1A, *p* < 0.05). At PD32, the growth-restricted rats provided either medium or high iron from PD2 to PD20 showed a significant increase in body weight compared with growth-restricted rats that were not provided iron (t-test, *p* < 0.05, Fig 1B). Together, these results suggest that there is a post-treatment effect of iron supplementation on growth, which is only significant in rats that consumed a calorie-restricted diet prior to weaning. It is possible that the growth trajectory of newborn rodents is less affected by iron than human infants. Although the weight differences found are small, we consider them to be of importance, especially considering the role of catch-up growth in poorer cognitive development in humans.

To evaluate the effect of the different iron treatments, hemoglobin, hematocrit as well as spleen and liver iron were measured at PD20 (Table 1). In general, these results are in agreement with our previous work using the same study design [15]. In normally fed pups, hemoglobin, hematocrit, liver and spleen iron were significantly higher in both the medium and high iron groups compared to controls. In the energy-restricted rats, hematocrit, liver and spleen iron were significantly elevated in response to both medium and high iron supplementation, but hemoglobin was only elevated in the medium iron group (t-test). There was no significant difference between the normal and growth-restricted controls in terms of hemoglobin or hematocrit levels, nor in liver and spleen iron (t-test), suggesting that energy restriction before weaning does not directly impact iron status.

**Table 1:** Hemoglobin, hematocrit and tissue iron content in liver and spleen at PD20.

	Normal			Growth Restricted		
	Veh (control)	30 μg/d	150 μg/d	Veh (control)	30 μg/d	150 μg/d
**Hg (g/L)**	**121.1 ± 2.4**	130.7 ± 2.9 *	134.3 ± 5.9 *	**119.9 ± 2.6**	135.6 ± 5.4*	**120.8 ± 6.8**
**HCT (%)**	**33.3 ± 0.7**	40.3 ± 0.9 *	40.0 ± 1.0 *	**27.5 ± 1.6**	34.0 ± 0.9 *	35.3 ± 1.1 *
**Liver Fe (μg/g)**	**54.3 ± 4.5**	100.6 ± 17.5 *	340.4 ± 56.9 *	**64.9 ± 8.2**	198.1 ± 16.3 *	538.4 ± 27.4 *
**Spleen Fe (μg/g)**	**139.3 ± 5.0**	179.7 ± 1.9 *	205.8 ± 18.7 *	**168.1 ± 15.7**	311.3 ± 51.7 *	391.6 ± 36.8 *

Hemoglobin, hematocrit, and tissue iron content in liver and spleen of rats at PD20. Values are presented as mean **±** SEM.

*p < 0.05, assessed by Student’s t-test compared to Veh (control).

### Cognitive development

**Fig 2:**
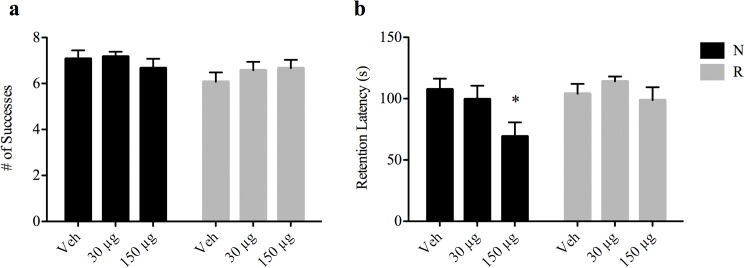
Effect of iron supplementation on cognitive development. (**a**) T-maze test scores at PD35. Scores between N-veh and R-veh groups were not significantly different (7.08 ± 0.36 successes vs. 6.08 ± 0.40 successes, *p* = 0.08) and no differences were found in scores between treatment groups. (**b**) Passive avoidance test at PD40. Data is expressed as time (s) taken to enter the dark chamber on d2 of the test, designated as retention latency. Latency was significantly decreased in N-150 μg pups compared to the Veh controls (107.54 ± 8.73 s vs. 69.14 ± 11.55 s, * *p* < 0.05, one-way ANOVA). Data are presented as means ± SEM.

To evaluate the impact of pre-weaning iron supplementation on post-weaning cognitive performance, a T-maze test was performed at PD35 and a passive avoidance test at PD40. There were no differences in T-maze scores between the iron treated groups and the respective controls for either normal or energy restricted rats (t-test, Fig 2A). Interestingly, in the passive avoidance test, introduction of high iron to normal rats significantly lowered the retention latency compared to control (t-test, *p* = 0.01, Fig 2B). No significant effect of iron on the passive avoidance score was observed in the growth restricted rats.

### Plasma metabolic profile

**Fig 3:**
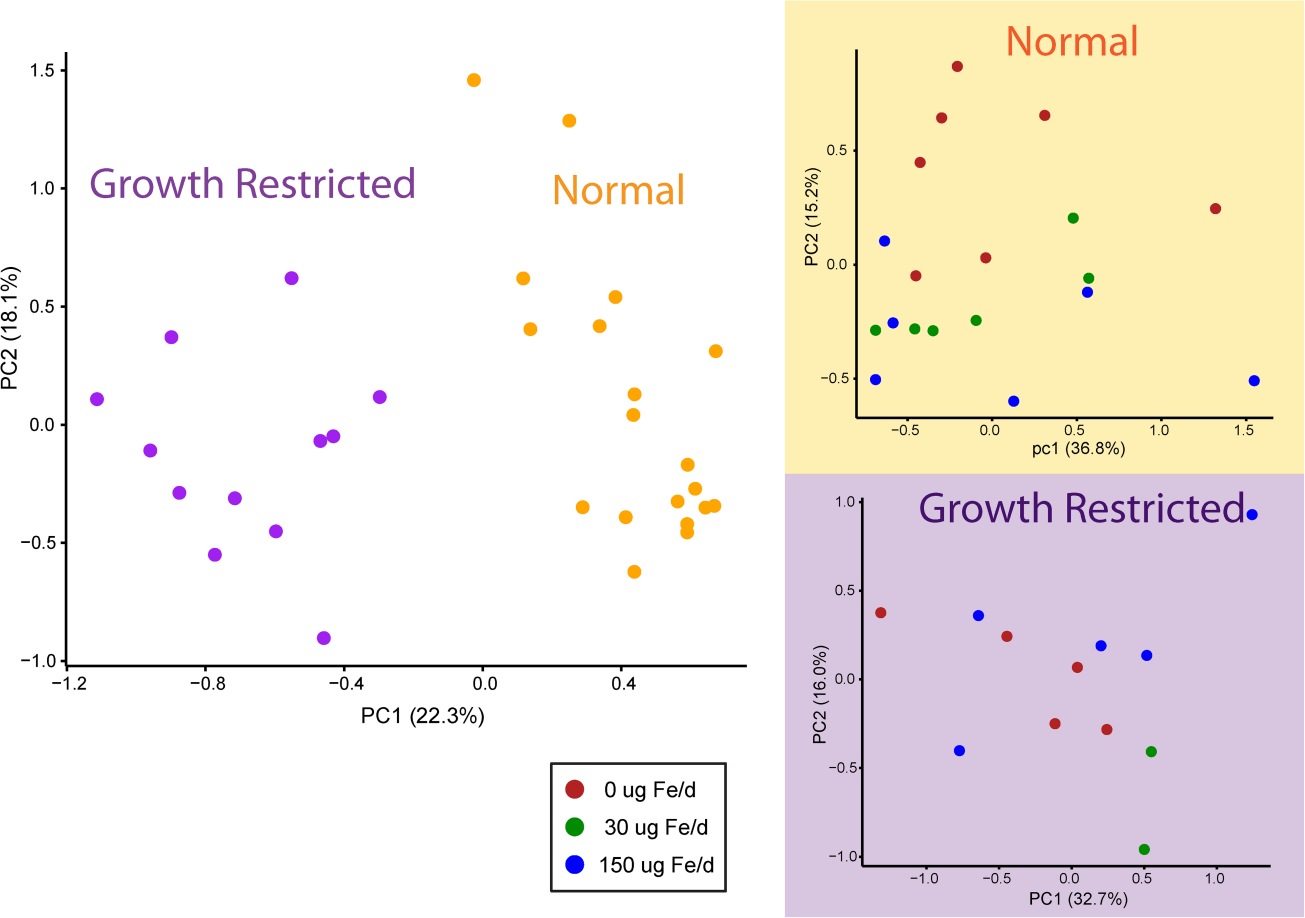
Principal component analysis of plasma metabolites at PD20. Principal component analysis of plasma metabolite variables from rat pups at PD20 from either a normal (orange) or growth restricted (purple) group. PC1 explains 22.3% of the variation, whereas PC2 explains 18.1%. Inset shows PCA of individual groups colored by amount of iron supplemented. 0 μg (red), 30 μg (green), 150 μg (blue) iron.

**Fig 4:**
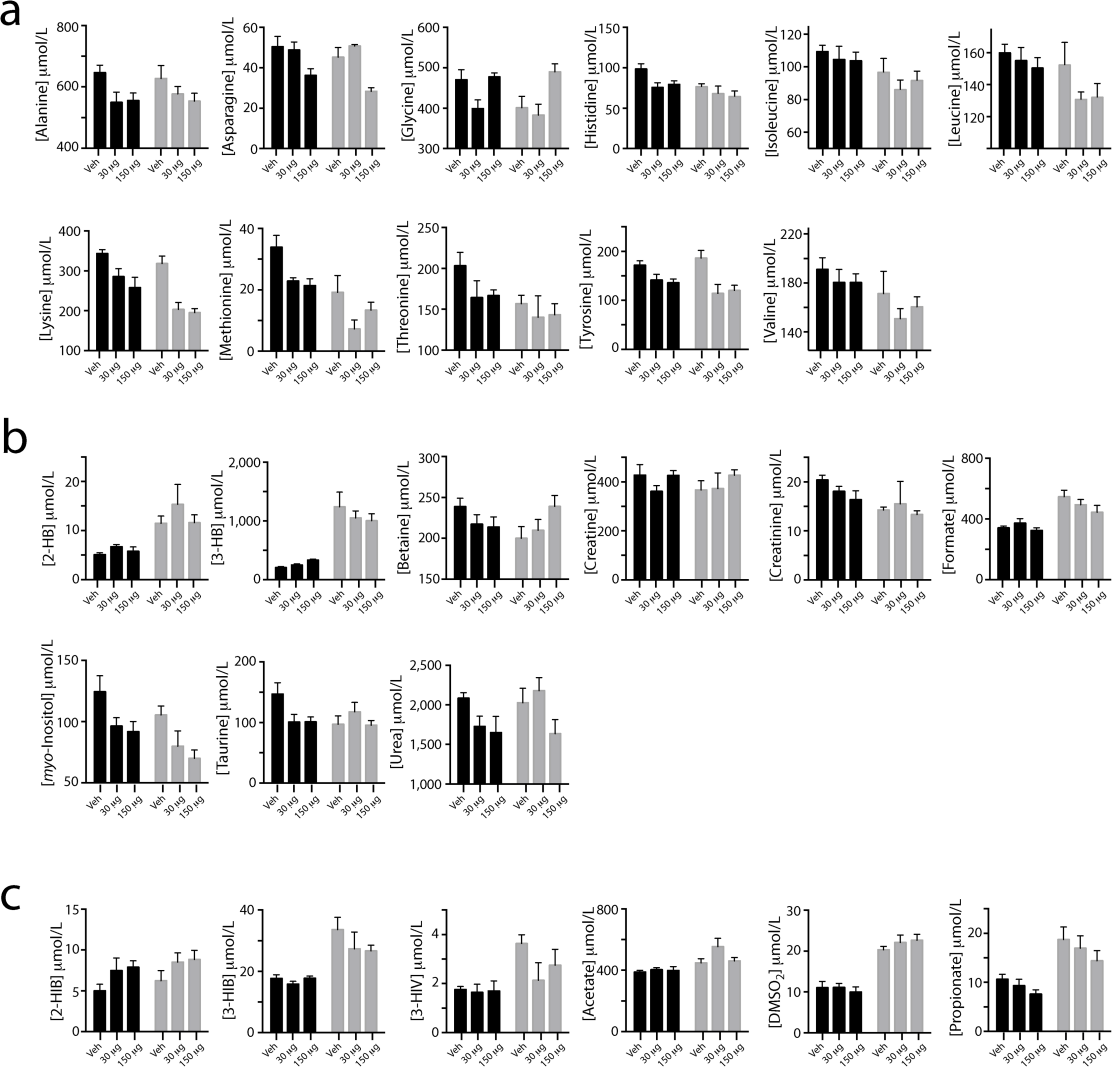
Plasma metabolite concentrations. Metabolite concentrations (mean ± SEM) in normal (black) and growth-restricted (grey) rats as a function of iron supplementation (Veh, 30 μg, or 150 μg per day). (**a**) Plasma amino acids. (**b**) Other plasma metabolites. (**c**) Metabolites of possible microbial origin. Abbreviations: 2-Hydroxybutyrate (2-HB); 3-Hydroxybutyrate (3-HB); 2-Hydroxyisobutyrate (2-HIB); 3-Hydroxyisobutyrate (3-HIB); 3-Hydroxyisovalerate (3-HIV), Dimethylsulfone (DMSO_2_).

To evaluate the metabolic consequences of iron excess, plasma samples at the end of iron supplementation period (PD20) were subjected to NMR-based metabolomics analysis to quantify a range of metabolites including amino acids, sugars, ketones, and organic acids. Five samples associated with low sample volumes (less than 100 μL) were detected as outliers in PCA and eliminated prior to data analysis. PCA of the plasma metabolome revealed that the major contributor to the metabolic profile was the effect of the diet (Fig 3). Caloric restriction resulted in decreased circulating amino acids (glycine, histidine, isoleucine, leucine, lysine, methionine, threonine and valine), markers of muscle mass turnover (creatine and creatinine) and *myo*-inositol, and increased ketone bodies (3-hydroxybutyrate), the stress marker 2-hydroxybutyrate, and microbial by-products (dimethyl sulfone, 3-hydroxyisobutyrate, 3-hydroxyisovalerate as well as short chain fatty acids (acetate, formate and propionate)) (two-way ANOVA, *p* < 0.05). In addition, betaine and taurine were significantly reduced when comparing normal and growth restricted rats without iron supplementation (t-test, *p* < 0.05) (Fig 4).

The metabolic responses to iron supplementation were different among rat pups who were normally fed or calorically restricted. In rats consuming sufficient energy from milk, iron supplementation resulted in increased 3-hydroxybutyrate, decreased circulating amino acids (alanine, histidine, lysine, methionine, tyrosine and taurine) as well as urea (one-way ANOVA, *p* < 0.05, Fig 4). Other amino acids such as asparagine and glycine as well as *myo*-inositol were moderately decreased, whereas 2-hydroxyisobutyrate increased with iron supplementation (one-way ANOVA, *p* < 0.08). Rats consuming sufficient calories presented a nearly linear change with respect to increasing iron dosage; however, in rats consuming insufficient energy from milk, except for betaine, glycine, asparagine, lysine, and myo-inositol, the metabolic response showed a trend toward decreased levels at medium, and maintenance or recovery to levels similar to the unsupplemented group at higher iron treatment. Betaine and glycine increased with increased iron supplementation, whereas asparagine, lysine, and myo-inositol decreased with iron supplementation (one-way ANOVA, *p* < 0.08, Fig 4).

### Hepatic metabolic profile

**Fig 5:**
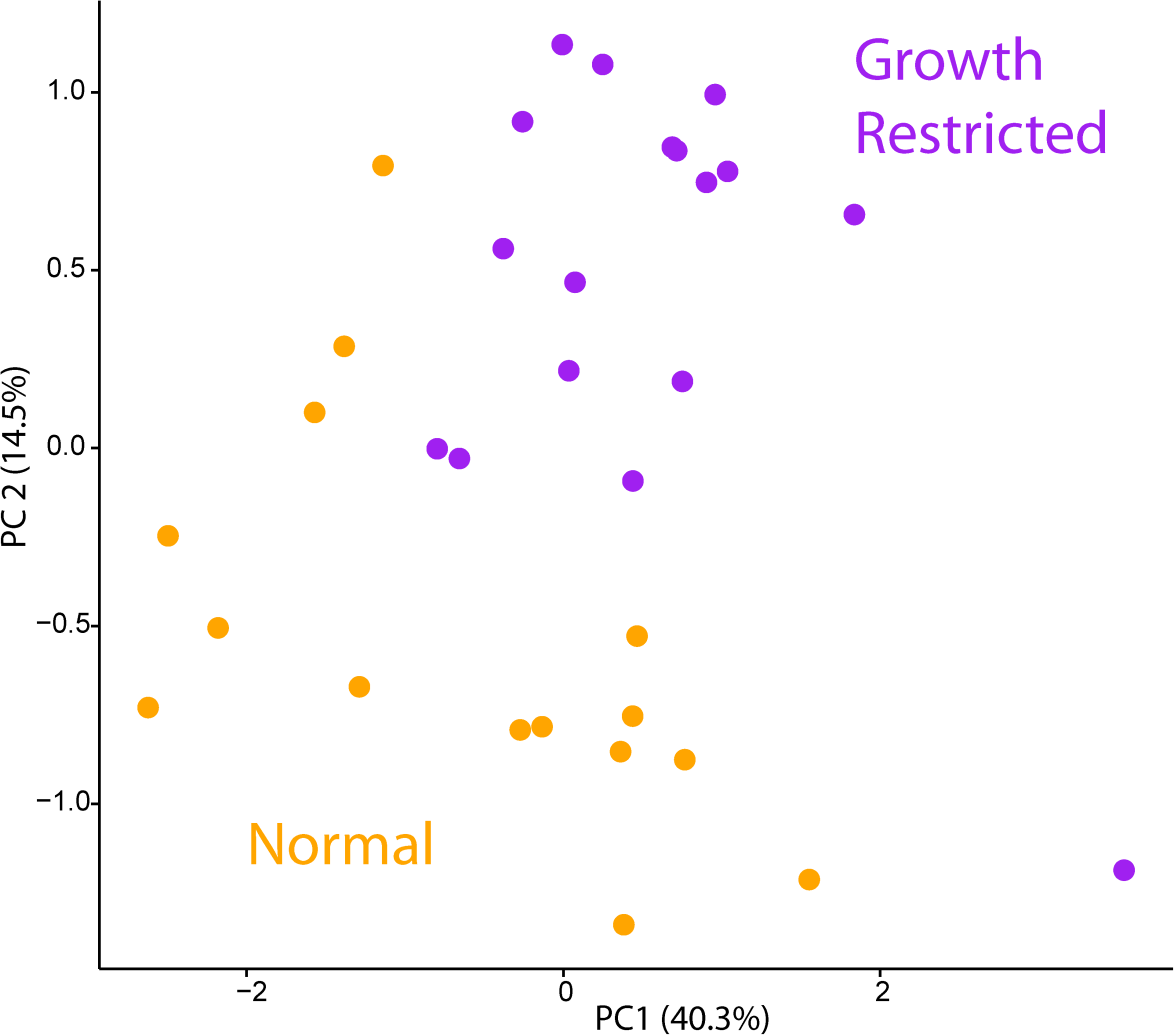
Principal component analysis of hepatic metabolites. Principal component analysis of hepatic metabolite variables from rat pups at PD20 from either normal (orange) or growth restricted (purple) group. PC1 explains 40.3% of the variation, whereas PC2 explains 14.5%.

Similar to the changes observed in plasma metabolites with growth restriction, the hepatic metabolic profile also revealed a profound difference between normal and energy- restricted rats (Fig 5). Specifically, energy restriction reduced hepatic amino acids levels (alanine, asparagine, glutamine, isoleucine, leucine, methionine, phenylalanine, tryptophan), *myo*-inositol, and increased ketone bodies (3-hydroxybutyrate) and short chain fatty acids (acetate and formate). Unlike circulating betaine (which was lower), hepatic betaine was significantly increased in growth-restricted rats. However, other methyl donors (S-adenosylhomocysteine, choline), choline’s precursor (phosphocholine), and an intermediate product of the choline oxidation pathway, sarcosine, were significantly reduced. The stress marker 2-hydroxybutyrate that was significantly increased in plasma was significantly reduced in the liver with growth restriction. Adipocyte fat catabolism products (glycerol and glycerol-3-phosphate), succinate, uridine and xanthosine were also found to be significantly reduced in the growth restricted rats compared to the control rats (two-way ANOVA).

Iron supplementation also induced a reduction of hepatic asparagine, xanthosine, and an elevation of formate in the control rats (one-way ANOVA, *p* < 0.05). Hepatic pyroglutamate, uridine and glycerol-3-phosphate were moderately reduced in control rats with iron supplementation (one-way ANOVA, *p* < 0.1). In contrast, only hepatic serine and uridine were reduced in the growth-restricted rats with iron supplementation (one-way ANOVA, *p* < 0.1). The concentration of glutathione in rat pup liver was too low to be measured.

### Gut microbiota

**Fig 6:**
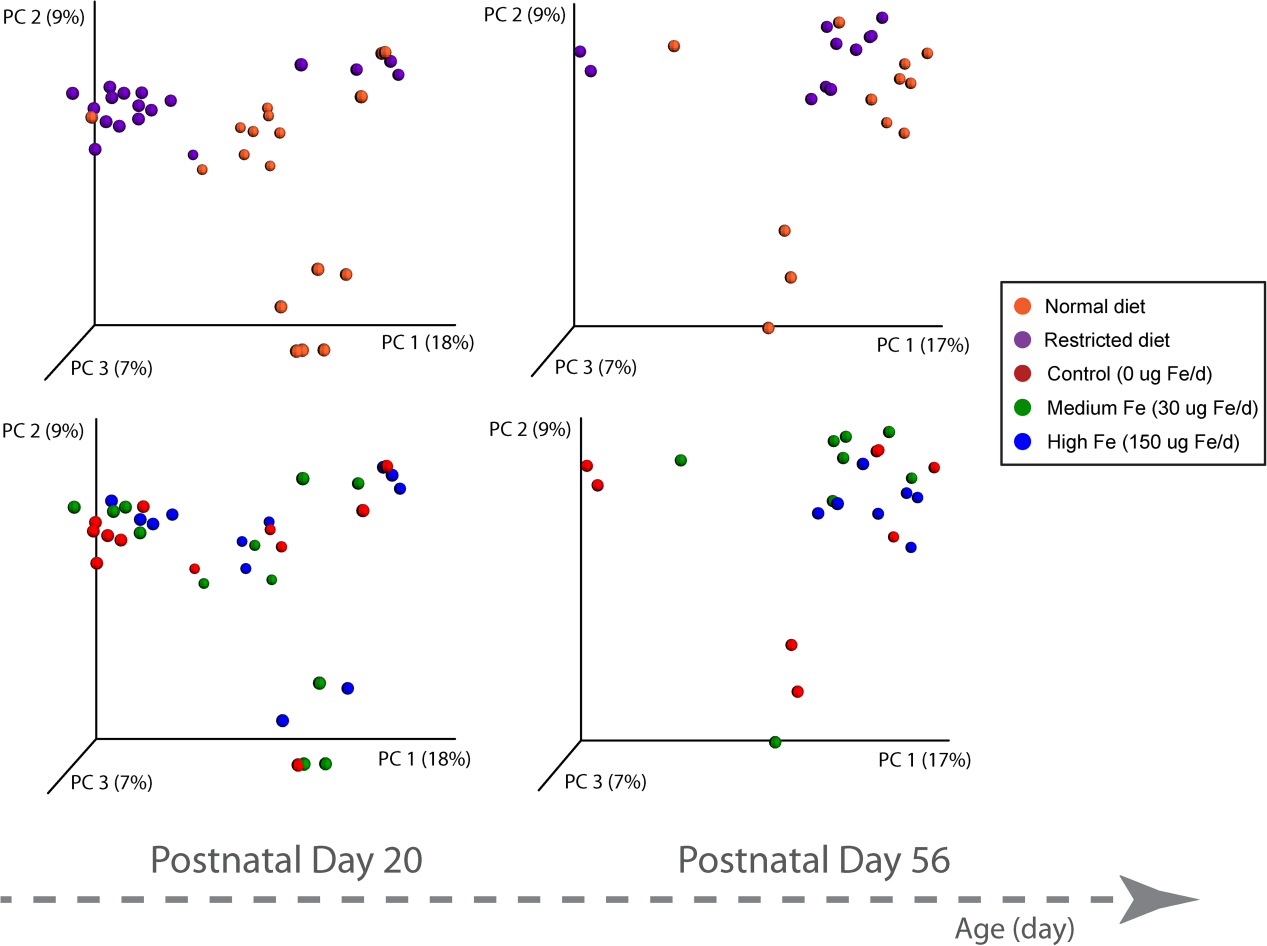
Principal coordinates analysis (PCoA) of 16s rRNA. PCoA of unweighted Unifrac distances of 16S rRNA sequences demonstrates clustering along PC1 based on diet (upper half of the figure) (purple represents restricted and orange as normal) for day 20 and 56. Clustering based on iron supplement (lower half of the figure) (0 μg (Control), 30 μg (medium), or 150 μg (high)).

**Fig 7:**
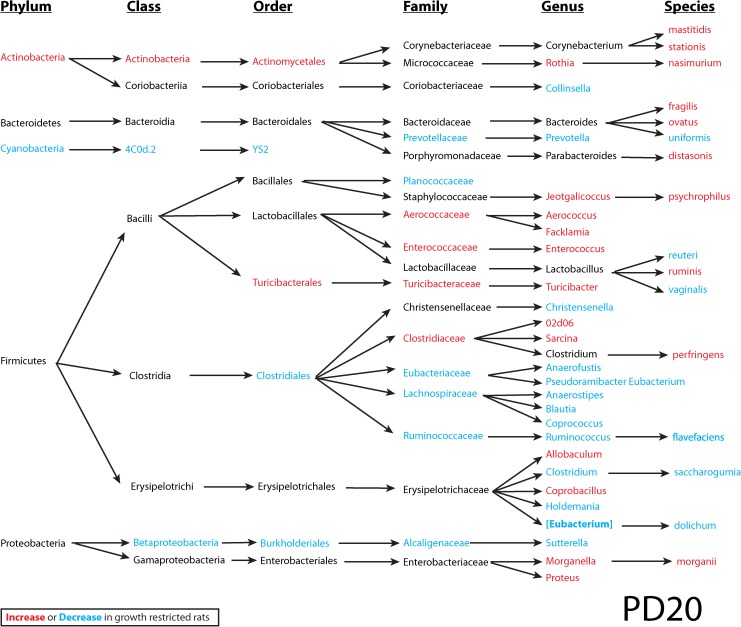
Colon microbial taxa at PD20. Significantly altered colon microbial taxa by diet at PD20. In red are taxa that increase, and blue are taxa that decrease in growth-restricted rats. The significance was determined using two-way ANOVA (iron treatment X diet) based on relative abundance data. The significance cut-off is *p* < 0.05. Disclaimer: in this small-scale pilot study (n = 4–6), multiple comparisons were not adjusted, and therefore results may contain false discoveries. Future confirmation is needed.

**Fig 8:**
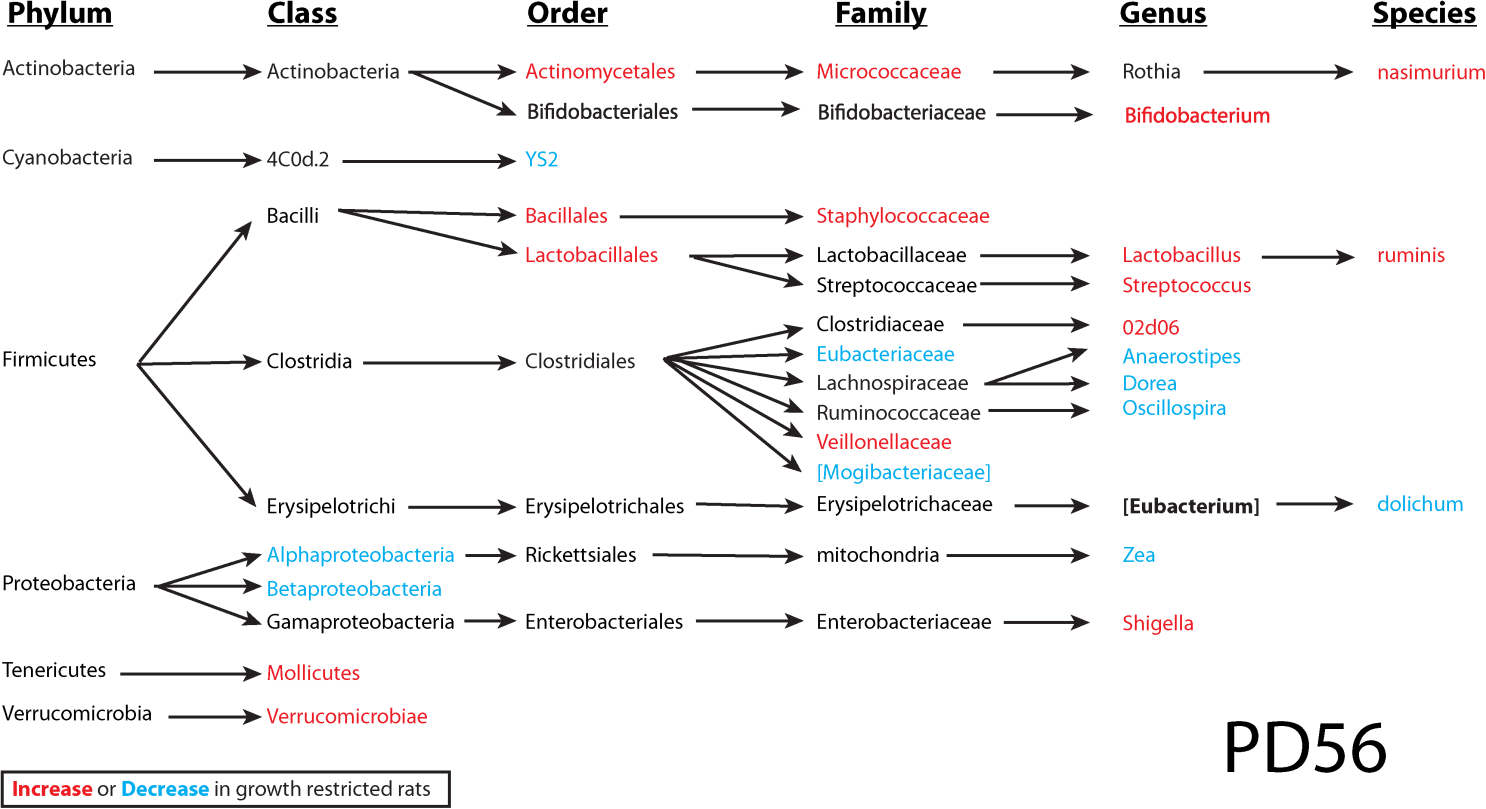
Colon microbial taxa at PD56. Significantly altered colon microbial taxa by diet at PD56. In red are taxa that increase, and blue are taxa that decrease in growth-restricted rats. The significance was determined using two-way ANOVA (iron treatment X diet) based on relative abundance data. The significance cut-off is *p* < 0.05. Disclaimer: in this small-scale pilot study (n = 4–6), multiple comparisons were not adjusted, and therefore results may contain false discoveries. Future confirmation is needed.

To investigate the interplay between dietary iron, the intestinal microbiota and the host, microbial 16S rRNA profiling of colon contents from rats at PD20 and PD56 was performed. At the end of the suckling period (PD20), a profound difference was observed between the normal and energy-restricted rats (Fig 6), indicating that diet plays an important role in shaping the gut microbiota. Differences based on litter were also observed in the gut microbiome data, contributing to the separation in PC2. Interestingly, although these pups were weaned onto solid food and fed *ad libitum* for an additional 36 days, the effect of diet was maintained at PD56, illustrating that early caloric restriction may have a long-lasting impact on gut microbiota. Analysis of bacterial OTU abundance at the phylum level revealed an increase in *Actinobacteria* and a reduction of *Cyanobacteria* in growth restricted rats compared to normal rats at PD20, which was not significant at PD56 (Figs 7 and 8). Because of the strong effect of diet and litter (primarily at PD20), an apparent effect of iron supplementation was not observed in this representation of the data. In comparison to previous observations on iron-induced microbiota of healthy infants and animals, elevated *Proteobacteria* and *Shigella* in normal rats consuming medium iron was observed at PD20, but not at PD56. In growth restricted rats, a reduction of *Bacteroidetes* was only observed at PD56 for those fed medium iron and a reduction of *Rothia* was observed at PD56 in both the medium and high iron group compared to the control. *Lactobacilli* were not altered by iron supplementation at PD20 in either normal or growth restricted rats; however, they increased at PD56 in growth-restricted rats that were provided medium iron as compared to controls (S1 Table). There was no significant effect of iron on bacterial diversity of observed species (one-way ANOVA followed by post-hoc Tukey test) in either normal or growth restricted rats at PD20 and PD56.

It is thought that the infant gut is first colonized by facultative anaerobes. While oxygen is gradually consumed, a more suitable environment for survival of strict anaerobes is created. As the gut matures, facultative anaerobes cannot resist competition, and they become outnumbered by strict anaerobes. In control rats, there were an increasing proportion of strict anaerobes from PD20 to PD56 (19.4% to 28.3%, t-test, *p* = 0.008). However, this was not significant in control rats given medium or high iron (*p* = 0.13, *p* = 0.80). Interestingly, at PD20, the proportion of strict anaerobes in the growth-restricted rats was similar to normal rats. With iron supplementation, a decrease in strict anaerobes (from 22.1% to 15.4%, *p* = 0.08 for medium iron and from 18.5% to 13.7%, *p* = 0.038 for high iron) was observed, suggesting that iron supplementation may be associated with elevated oxygen content in the gut that may lead to a delay in gut maturation.

## Discussion

Although it has been shown that iron supplements administered to iron-replete breast-fed infants can have a negative effect on linear growth or weight gain [4, 5, 7, 8], we did not find an effect on normally fed rat pups [15], or growth restricted rat pups. Linear growth is difficult to assess in young rats, but it is possible that the growth trajectory of the newborn rodent is less affected by iron than human infants. It should be noted that the daily amounts of iron provided are quite modest and far from what can be expected as toxic levels. The daily amount of iron provided in this study was based on body weight, and similar to the amount provided to infants in iron drops or iron-fortified formula. Though there are limitations to using rat pups to study human infants, both young rat pups and human infants lack the capacity to homeostatically regulate iron absorption [27]. Due to this absence of regulation, newborn rodents and human infants are able to absorb large proportions of dietary iron, especially during supplementation. Developed regulation of iron absorption does occur as rats and humans grow older, though when this programming occurs varies between species. Certainly, the differences in timing of development in rats versus humans must be taken into account.

Hemoglobin, hematocrit, liver and spleen iron significantly increased in the rat pups supplemented with iron. This result is in agreement with our previous work [15] showing that young rat pups lack the capacity to homeostatically regulate iron absorption. Our results are also consistent with a study by Unger et al. in which rat pups were fed excess iron [28]. Together, these studies demonstrate that newborn rodents are able to absorb a large proportion of dietary iron from supplements. We have previously shown, using stable isotopes of iron, that breast-fed infants lack the capacity to regulate iron absorption homeostatically [29], but that this develops when they grow older. Thus, infancy appears to be a period when iron is absorbed very well and mechanisms to regulate excessive iron accumulation are lacking or very limited. When provided iron supplements, the potential for adverse effects exists.

There was a latent effect by iron introduced before weaning on cognitive development in normally fed rats as assessed by the passive avoidance test. This test has been widely used to examine both short- and long-term memory and learning, and is well recognized to reflect hippocampal function [30, 31]. This behavior aspect of memory is similar in animals and humans [32]. It is interesting to note that Lozoff et al. found adverse effects on several measures of cognition, behavior and motor development in Chilean infants with adequate iron status who were given high iron infant formula [6]. These infants likely had adequate nutritional status and our results therefore suggest that this is a valid model for the human infant.

Several previous studies in rodent models have shown adverse effects of iron excess during infancy on neurological outcomes. Kaur et al. [33] showed that mouse pups given daily iron supplements from PD10-17 at a level similar to that provided to human infants fed iron-fortified formula showed increased nigrostriatal iron, and displayed progressive mid-brain neurodegeneration. Fredriksson et al. [34] showed that mouse pups given iron orally from PD10-12 had an almost complete lack of habituation of spontaneous activity at 3 months of age, and that the effect was dependent on the dose of iron given. Analysis of brain iron showed accumulation in the basal ganglia. They also showed that the effect was less pronounced when iron was given on PD1-3, and that there was no effect when it was given on PD19-21 [35]. These observations may be correlated to our observations on Swedish infants who showed no homeostatic regulation of iron absorption during “mid-infancy”, i.e. at 4–6 months of age, but down-regulation of iron absorption in older infants (6–9 months old) [29]. Thus, there may be a window of vulnerability during infancy when infants exposed to excess iron can absorb the iron, accumulate it in tissues and suffer negative consequences for neurodevelopment.

Normally fed and growth restricted rats had similar metabolic responses to medium iron supplementation, whereas the metabolic response of growth-restricted rats showed a larger difference between medium and high iron treatment (Fig 4). Growth restriction due to reduced energy intake may lead to low protein storage and a ketogenic state. Our results indicate that there are decreasing levels of circulating amino acids, markers of muscle mass turnover (creatine and creatinine), and *myo*-inositol, with increasing level of ketone bodies (3-hydroxybutyrate) and the stress marker (2-hydroxybutyrate) in the growth restricted pups.

Iron induced markers included 2-hydroxyisobutyrate, 3-hydroxybutyrate, alanine, betaine, dimethylamine, histidine, and taurine, which were highly sensitive to diet, implying that nutritional status and dietary intake should be carefully controlled when evaluating the metabolic effects of excess iron. Interestingly, we observed an iron-induced decrease in plasma methionine, and a moderate reduction of taurine and *myo*-inositol in the normally fed pups. Myo-inositol and taurine are thought to be important brain osmolytes [36, 37]. Furthermore, taurine can also act as a neurotransmitter and is known to be critical for infant brain development [38]. It is thus hypothesized that iron induced oxidative stress triggers elevation of hepatic glutathione peroxidase, which in turn reduces glutathione. Unfortunately, hepatic glutathione concentrations were too low to be measured in this study; however, circulating methionine and taurine were both significantly reduced, especially with medium iron supplementation.

The effect of diet on the hepatic metabolome is in agreement with the result from the plasma metabolome, with decreased amino acids, *myo*-inositol and increased ketone bodies and short-chain fatty acids. Hepatic asparagine and xanthosine were also decreased, whereas formate was elevated by iron supplementation. Asparagine is known for the generation of nitric oxide (NO), an important messenger molecule in several key biological functions including neurotransmission, regulation of blood pressure and immune response [39]. NO as a neurotransmitter plays an important function in learning and memory [36]. Although the exact mechanism of iron oral supplementation on NO metabolism remains to be investigated, it has been shown that in the presence of LPS, iron administration to rats intraperitoneally leads predominantly to iron uptake by liver Kupffer cells which is associated with an increased NO level in the blood [40, 41]. Further studies are needed to confirm the relationship between asparagine, NO and iron overload.

Diet is the primary driver over other factors shaping gut microbiota [42]. In the present study, diet was the major contributor in determining the gut microbiota profile, followed by the litter effect. Iron supplementation alone contributes a minor effect toward the overall gut microbial composition and diversity. It has been hypothesized that increased unabsorbed dietary iron due to supplementation may modify the intestinal microbiota and promote growth of pathogenic strains. However, many of these studies on gut microbiota were on populations in poor sanitation areas and with an elevated risk of pathogen or helminth infection [13, 43]. For example, iron supplementation of children who consumed a low-quality diet and lived in an African rural area with poor sanitation, showed reduced *Lactobacilli* and elevated *Enterobacteriaceae* [43]. In contrast, most studies on the adverse effects of iron excess on infant/child growth/cognitive development have focused on more developed countries with better hygiene [5, 6, 9, 10]. In the present study, we did not observe a profound impact of iron supplementation on individual bacterial OTUs, which contradicts previous studies (summarized in S1 Table). Nonetheless, our results are in agreement with the study by Dostal et al. that found no effect of iron supplementation on the fecal microbial profile of South African children who only had mild iron deficiency, were in a malaria-free environment, and lived in households with access to clean tap water [44]. It is likely that environment plays a key role in bacteria affected by excess iron.

It is well known that microbial plasticity exists during infancy [45]. While the functional role of individual gut bacteria OTUs, as well as how the microbial composition shifts over time still need to be explored, one mechanism to explain these changes could be that higher levels of free reactive Fe (II) may induce free radical damage to the gastrointestinal tract and subsequently release oxygen as a by-product of the Haber-Weiss reaction [46], causing oxidative stress which then reduces the level of strict anaerobes to slow maturation to an “adult-like” gut microbial profile. In this study, intestinal ROS was not directly measured, and the glutathione level was below our limit of detection. However, there are a few indirect findings that support our hypothesis. First, the iron-supplemented rats no longer exhibited an increased proportion of strict anaerobes from PD20 to PD56, whereas the normal succession of gut microbiota may be characterized as a migration from more facultative organisms to strict anaerobes in the gut. Secondly, the oxidative stress protective metabolites that include methionine [47] and taurine [48] were significantly decreased in the iron supplemented rats. Further studies involving the direct measurement of ROS and oxidative stress enzymes in other tissues such as the brain and intestine are needed to elucidate this.

There are several limitations of using neonatal rat pups as a model to study human infant development. Although rodent models have the advantage of a relatively uniform genetic background, the anatomy of the rodent and human intestinal tract is different. The rodent has a large cecum where the majority of fermentation occurs, whereas the human cecum is relatively small and the colon is the major fermentation site (reviewed in [49]). Unlike human infants, rodent pups are born very immature with respect to gut and brain development. The gut of newborn pups matures gradually during nursing (from birth to day 21), and a particularly rapid maturation takes place during the short period of transition from milk to solid food (weaning). The intestinal tract of human infants is more mature at birth (reviewed in [50]). Furthermore, there is a clear difference in the level of specific genera/species abundance of the gut microbes in human infants compared with the neonatal rat. For example, a high relative abundance of *Lactobacillus* (on average 54.3% and 57.6% in PD 20 and PD 56 respectively) was observed in rat pups, whereas the number of *Bifidobacteria* was always below 0.08% in relative abundance. In comparison, the *Bifidobacterium* level is much higher in human infants (from ~22% at day 8 to ~40% at Day 120), which may be correlated with the presence of 2’-fucosylated glycans in human milk [51]. Other confounding factors such as coprophagy, maternal inoculation, and housing conditions can also influence the composition of the rodent gut microbiota [49]. In this study, rat pups were housed together with their dams during the treatment period (PD2 to PD20), which appeared to introduce litter effects on the microbiome due to the shared environment. Because of the pilot scale of this project, we did not adjust p-values for multiple comparisons in both the microbiome and metabolome data sets, and therefore some results may be false discoveries.

In summary, our results show that excess iron supplementation during the early postnatal period improved the growth of growth-restricted animals, while adversely affecting memory and learning in iron-replete animals. Changes in the plasma and liver metabolomes suggest that iron supplementation has an effect on metabolism. Greater differences were observed in the microbiome with dietary restriction than with iron supplementation, but iron supplementation did impact the number of strict anaerobes. While the optimal amount of iron to be administered as a supplement continues to be debated, it is clear that the adverse effects of excess iron must be considered as well. These findings support the use of this model to study the mechanisms behind the adverse effects of excessive iron supplementation.

## Supporting information

S1 Table Summary of ferrous sulfate supplementation studies. (PDF)
