# Supplementary material for: Effects of iron supplementation on growth, gut microbiota, metabolomics and cognitive development of rat pups
Source: PLoS One. 2017 Jun 29;12(6):e0179713. doi: 10.1371/journal.pone.0179713 (PMC5491036; doi:10.1371/journal.pone.0179713)
Supplement: S1 Table — (PDF) [file pone.0179713.s001.pdf]

Ferrous sulfate supplementation studies

Study	Zimmermann et al [1]	Jaeggi et al [2]	Krebs et al [3]	Dostal et al [4].	Tompkins et al [5]	Benoni et al [6]	Lee et al [7]	Current study			
Subject/animal	African children (n=139). 54% of children were infected with helminths, live in remote rural area with poor sanitation and low quality diet	Kenyan infants (n=115). Malaria-endemic area, 67.3% were anaemic, 25.5%, 99% breast-fed, 80% already been introduced to complementary food	Breast-fed term infants (n=55). 27% had low ferritin level, 36% had mild anemia.	South African children (n=73), from households with access to clean tap water, malaria-free environment, only mildly Fe deficient.	Male Swiss-Webster mice	Sprague Dawley rats	Weaning piglets (n=144)	Normal rat pups		Growth restricted rat pups	
								PD 20	PD 56	PD 20	PD 56
Iron form	Iron-fortified biscuit, 8.8 mg Fe/d from supplementation. 12 mg Fe/d from daily meal.	Micronutrient powder containing NaFeEDTA, ferrous fumarate, daily	Pureed meats compare to organic, whole grain iron-only fortified infant cereal.	50 mg Fe as ferrous sulfate for 4 d/week	121 mg or 1.59 g Ferric chloride/kg compared to the control group supplemented with 2mg iron/kg from base diet with NaCl	Ferrous sulfate, 10 and 30 mg iron/kg	50, 100 or 250 mg iron as ferrous sulfate /kg	Daily supplementation of medium (30 µg) or High (150 µg) iron as ferrous sulfate			
Age	6-13 y old	6 month old	5 months old	6-11 y old	32 d old		birth to 28 days	Weaning (from birth to age of 56 days)			
Duration	6 months	4 months	4-5 months	38 weeks	8 weeks	2 or 4 weeks	28 days	From day 2 to day 20 after birth			
Gastrointestinal illness	Diarrhea, NC. Constipation, reduced, vomiting, increased.	Diarrhea, increased. Malaria, NC.		Diarrhea, stomach pain, vomiting, NC.			Diarrhea, increased				
Method	Temporal temperature gradient gel electrophoresis and qPCR	16S pyrosequencing, qPCR	16S pyrosequencing	qPCR	Culture-based	Culture-based	Culture-based	Illumina 16S sequencing			
**Firmicutes**		Increased		NC				NC	NC	NC	NC
Enterococcus					Reduced	Reduced		NC	NC	NC	NC
Bacilli			Reduced					NC	NC	NC	NC
Lactobacillus	Reduced		Reduced	NC	Reduced	Increased after 2 weeks	NC	NC	NC	NC	Increased in medium Fe
Clostridium		Increased	Clostridium Cluter XIVa decreased	Clostridium Cluster IV and Eubacterium hallii,, NC		Increased		NC	NC	Increased in High Fe (1)	NC
Peptostreptococcus						Reduced at 4 weeks, increased after 2 weeks.		ND	ND	ND	ND
**Bacteroidetes**			Increased	NC		Reduced after 4 weeks, increased after 2 weeks		NC	NC	NC	Reduced in medium Fe
**Actinobacteria**			Reduced					NC	NC	NC	NC
Bifidobacterium	NC	Reduced	Reduced	NC			NC	NC	NC	NC	NC
Rothia			Reduced					NC	NC	NC	Reduced in both median and High Fe
**Proteobacteria**			NC					Increased in medium Fe (2)	NC	NC	NC
Enterobacteriaceae	Increased		Decrease (10.3% vs. 23.1%)	NC				NC	NC	NC	NC
Shigella	Increased	Increased	NC				Coliform bacteria, NC	Increased in medium Fe (3)	NC	NC	NC
E.coli			NC			Increased		NC	NC	NC	NC

* One-way ANOVA followed by HSD turkey post-hoc test. NC = No change. ND = Not detected.
(1) In normal diet, Clostridium from family Clostridiaceae is significantly increased in high Fe group compared to the control. This does not include Clostridium from Lachnospiraceae and Erysipelotrichaceae family.
(2) p=0.07 using pairwised post-hoc Tukey test.
(3) p=0.06 using pairwised post-hoc Tukey test.

[1] Zimmermann MB, Chassard C, Rohner F, N'Goran E K, Nindjin C, Dostal A, et al. The effects of iron fortification on the gut microbiota in African children: a randomized controlled trial in Cote d'Ivoire. The American journal of clinical nutrition. 2010;92:1406-15.
[2] Jaeggi T, Kortman GA, Moretti D, Chassard C, Holding P, Dostal A, et al. Iron fortification adversely affects the gut microbiome, increases pathogen abundance and induces intestinal inflammation in Kenyan infants. Gut. 2015;64:731-42.
[3] Krebs NF, Sherlock LG, Westcott J, Culbertson D, Hambidge KM, Feazel LM, et al. Effects of different complementary feeding regimens on iron status and enteric microbiota in breastfed infants. J Pediatr. 2013;163:416-23.
[4] Dostal A, Baumgartner J, Riesen N, Chassard C, Smuts CM, Zimmermann MB, et al. Effects of iron supplementation on dominant bacterial groups in the gut, faecal SCFA and gut inflammation: a randomised, placebo-controlled intervention trial in South …. Br J Nutr. 2014;112:547-56.
[5] Tompkins GR, O'Dell NL, Bryson IT, Pennington CB. The effects of dietary ferric iron and iron deprivation on the bacterial composition of the mouse intestine. Curr Microbiol. 2001;43:38-42.
[6] Benoni G, Cuzzolin L, Zambreri D, Donini M, Del Soldato P, Caramazza I. Gastrointestinal effects of single and repeated doses of ferrous sulphate in rats. Pharmacol Res. 1993;27:73-80.
[7] Lee SH, Shinde P, Choi J, Park M, Ohh S, Kwon IK, et al. Effects of dietary iron levels on growth performance, hematological status, liver mineral concentration, fecal microflora, and diarrhea incidence in weanling pigs. Biol Trace Elem Res. 2008;126 Suppl 1:S57-68.
